# The real taxonomic identity of *Trigona latitarsis* Friese, 1900, with notes on type specimens (Hymenoptera, Apidae)

**DOI:** 10.3897/zookeys.713.11653

**Published:** 2017-11-02

**Authors:** David Silva Nogueira, Favízia Freitas de Oliveira, Marcio Luiz de Oliveira

**Affiliations:** 1 Laboratório de Hymenoptera, Coordenação de Biodiversidade, Instituto Nacional de Pesquisas da Amazônia, Av. André Araújo, 2936 – Petrópolis, CEP 69067-375, Manaus, Amazonas, Brazil; 2 Laboratório de Bionomia, Biogeografia e Sistemática de Insetos – BIOSIS, Instituto de Biologia, Universidade Federal da Bahia, Rua Barão de Jeremoabo, s/n. Campus Universitário de Ondina, Salvador, CEP 40170-115, Bahia, Brazil

**Keywords:** Identification, lectotype, Meliponini, morphology, stingless bee, taxonomy

## Abstract

The taxonomic history of *Trigona latitarsis* Friese, 1900 and its clarification based on the observation of the types and literature data are treated in this study. The paper discusses the validity of the previously proposed lectotype, deposited in the ZMB (Berlin, Germany). Based on the type series deposited in HNHM (Budapest, Hungary) as well as the original description, a new lectotype and 15 paralectotypes from Amazon forest (São Paulo de Olivença, Amazonas) are designated. Data on the geographic distribution of *Scaura latitarsis* (Friese, 1900) are provided.

## Abstract (pt)

A história taxonômica de *Trigona latitarsis* Friese, 1900 é tratada nesse estudo, bem como esclarecimentos com base na observação dos tipos e em dados de literatura. É discutida a validade do lectótipo proposto anteriormente e depositado na ZMB (Berlim, Alemanha). Com base na série-tipo depositada no HNHM (Budapeste, Hungria), conforme descrição original, lectótipo e paralectótipos da floresta Amazônica (São Paulo de Olivença, Amazonas) são designados. Dados sobre a distribuição geográfica de *Scaura latitarsis* (Friese, 1900) são também fornecidos.

## Introduction

The genus *Scaura* Schwarz, 1938 is composed of small and dark bees, with unusually enlarged hind basitarsus (wide as or wider than the hind tibia). The genal area is narrower than the compound eye in profile view and the malar space shorter than the diameter of the flagellum (Silveira et al. 2002). The distribution of the genus is broadly Neotropical, from Mexico to southern Brazil (Camargo and Pedro 2013).

*Scaura* was first described by Schwarz (1938) as a subgenus of *Trigona* Jurine, 1807, under the name Trigona (Scaura) latitarsis Friese, 1900, based on the enlarged hind basitarsus as the main diagnostic characteristic. Later, Schwarz (1948) included *Melipona longula* Lepeletier, 1836, in this subgenus, considering it to have two subspecies (originally treated as varieties): T. (S.) longula longula and T. (S.) longula tenuis (Ducke, 1916), based on width of the hind basitarsus, the edentate mandible and the rather triangular hind tibiae. Currently, these three forms are considered distinct species (Michener 1990, Camargo and Pedro 2013, Oliveira 2016), included in *Scaura* as an individualized genus apart from *Trigona*. This interpretation was initiated by Moure (1944) when he included three species of *Scaura* in a list of Peruvian species. Additional recognition characters of *Scaura* were provided by Moure (1951) who highlighted the absence of yellow spots on body, the malar space narrower than the flagellum diameter, the forewing with acute angle in submarginal cell and the abdomen narrower than the thorax.

In the original description of *Trigona latitarsis*, Friese (1900) described the female and the male, citing three workers and three males among the type material from Brazil (São Paulo) and Suriname. However, he does not identify the institution in which they were deposited, but he included several males from São Paulo among the syntypes that are deposited in the Hungarian Natural History Museum (HNHM - Budapest, Hungary). The specimen designated by Melo and Costa (2004) as lecotype of *Trigona latitarsis* is a worker from Jundiaí, São Paulo, which is deposited in the Museum für Naturkunde Berlin (ZMB, Berlin, Germany).

Based on some doubts arising from the study of the type material of *Trigona latitarsis*, the aim of the present study is to clarify some issues related to the taxonomic identity of *Scaura latitarsis*, based on the information available in the original description and of records species occurrence. The study also discusses the validity of the lectotype designated by Melo and Costa (2004), adds information on the syntypes, the type locality, morphological characters of the type series specimen, its taxonomic identify and, finally, the designation of a specimen of the type series as the new lectotype.

## Materials and methods

The type material designated by Friese was studied by the second author of this study, during her trip to the HNHM in 2014. All the specimens deposited in those collections which were identified and labeled by Friese as *Trigona latitarsis* were studied. Specimens found and identified as belonging to the type series of *Trigona latitarsis* were borrowed and brought to Laboratório de Bionomia, Biogeografia e Sistemática de Insetos (BIOSIS-UFBA, Salvador, Bahia, Brazil). There, they were studied, measured, and photographed with the use of a stereoscopic microscope Leica M165C, coupled with a digital camera Leica DFC295 and containing the Application Suite V4.1 Interactive Measurements, Montage program.

**Figures 1–4.**
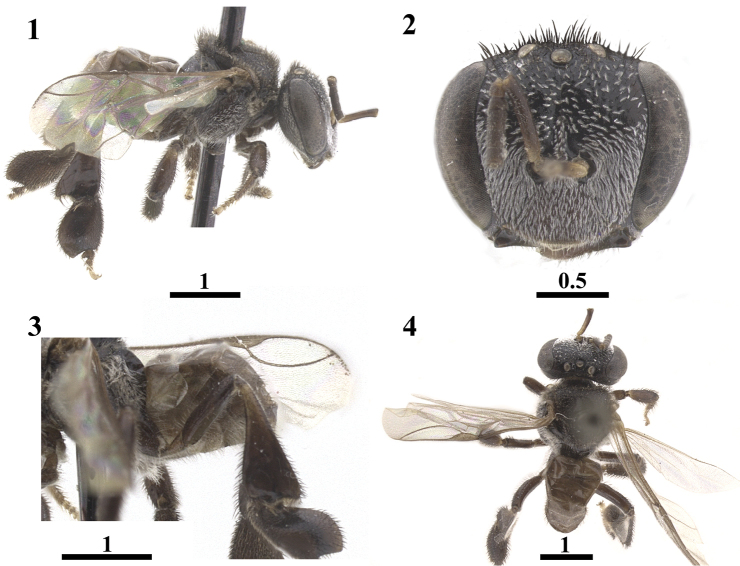
Lectotype of *Trigona latitarsis* Friese, 1900, designated by Melo and Costa (2004) deposited at the ZMB, invalidated in this study. Lateral view **1** Frontal view of head **2** Lateral view of hind leg and metasoma **3** Dorsal view **4** Scale bars in millimeters.

The material designated by Melo and Costa (2004) as lectotype of *Trigona latitarsis* (Figs 1–4) was loaned from Museum für Naturkunde Berlin (**ZMB**, Berlin, Germany) to the Laboratório de Hymenoptera, Instituto Nacional de Pesquisas da Amazônia (**INPA**, Manaus, Amazonas, Brazil), where it was studied with the use of the same stereomicroscope described above.

In the diagnosis below, the symbol “♀” refers to worker, “♂” refers to male, “**Q**” to queen, “**S**” to sterna and “**T**” to terga.

## Taxonomic characterization

### Scaura latitarsis

Taxon classificationAnimaliaHymenopteraApidae

(Friese, 1900)

Figs 5–8 , 9–12 , 13–18 , 19, 20 , 21–24 , 25 , 26

Trigona latitarsis Friese, 1900: 388. Lectotype male (presently designated). Label data: written with black ink: “Brasil, S. Paulo, 631/156”.
Melipona crassipes tenuis Ducke, 1916: 46, 47; Ducke, 1925: 342, 368 [taxonomic characters, geographic records]
Scaura crassipes tenuis ; Moure 1944: 28, 29 [list].
Trigona (Scaura) longula tenuis ; Schwarz 1948: 489, 499, 500 [taxonomic characters, diagnosis, key, geographic records].
Trigona (Scaura) tenuis ; Wille and Michener 1973: 12.
Plebeia (Scaura) tenuis ; Michener 1990:114 [systematics, taxonomic characters, key]; Nates-Parra 2001: 239 [geographic records]; Smith-Pardo, 2003: 388 [list].
Scaura tenuis ; Silveira et al. 2002: 91 [list, geographic records]; Camargo and Pedro 2002: 108 [taxonomic characters]; Camargo and Pedro 2013 [online catalog]; Oliveira et al. 2013: 163–167, 228. figs 297–313 [taxonomic characters, geographic records, key]; Oliveira 2016 [online catalog]. **Syn. n**.

#### Diagnosis.

**Figures 5–8.**
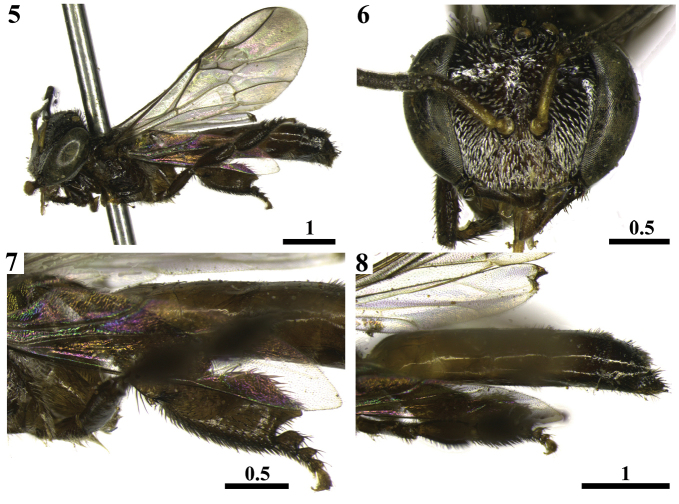
Paralectotype worker of *Trigona latitarsis* Friese, 1900 deposited at the Entomological Collection of the HNHM. Lateral view **5** Frontal view of head **6** Lateral view of hind leg **7** Lateral view of metasoma **8** Scale bars in millimeters.

**Figures 9–12.**
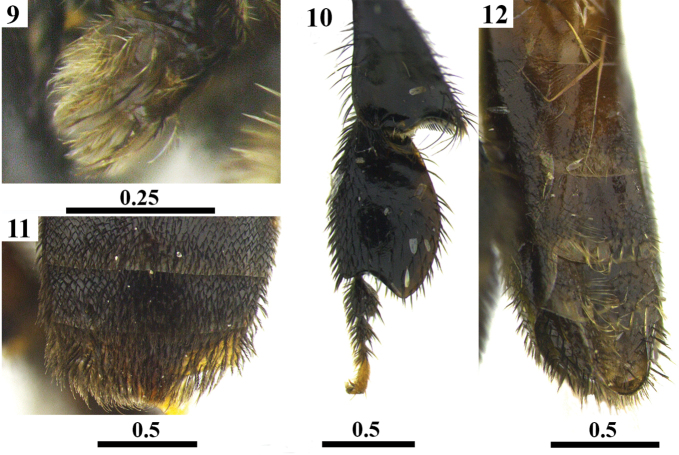
Details of *Trigona latitarsis* Friese, 1900 workers of the additional material examined from Amazonas State. Blackish setae among the ramified whitish setae on pronotal lobe **9** Apex of the posterior border of mesothoracic basitarsus sharp **10** Blackish setae on T4 to T6 **11** Whitish setae on sterna **12** Scales in millimeters.

(♀) Scape almost entirely yellow, with a dark spot on the apical third of dorsal surface (Fig. 6); forehead setae blackish and hairs whitish mixed; whitish simple and ramified hairs on mesosoma; blackish setae intercalating the ramified whitish setae on pronotal lobes (Fig. 9); apex of the posterior border of mesothoracic basitarsus sharp (Figs 5, 10); metasoma elongated; blackish setae on T4 to T6 and whitish on sterna (Figs 11, 12). (♂) Ocellocular distance similar in length to the diameter of the lateral ocellus; blackish setae on vertex; whitish hairs on mesosome, blackish setae in terga T4 to T6 and whitish on sterna; valves with rounded base and tapering gradually to apex; posterior groove of spathe deep and sharp in dorsal view (Fig. 19); gonocoxite similar to an equilateral triangle; setae short and thick, restricted to the apical half of inner face of gonostile and the fourth apical of outer face. (Q) Setae of mandibles about half the mandible length; propodeum slightly concave in lateral view; metathoracic basitarsus about 5.5× longer than broad; junction area of fifth sternum apical lobes straight in ventral view; separation of these lobes a little bit pronounced.

#### Worker redescription.

***Paralectotype worker* (Figs 5–12).** *Color*: Integument predominantly brown-blackish. Clypeus dark brown, labrum yellowish brown. Mandibles brown, except the yellow apical 1/4. Mandibular condyles more darkened. Scape yellowish, with a brown spot in the apical third of dorsal surface; flagellum brown. Pronotal lobe yellowish-brown. Legs light brown, coxae slightly lighter than the other segments. Femora, tibiae and basitarsus slightly darkened on sides. Two last prothoracic tarsomeres yellowish-pale. Last segments of meso and metathoracic legs yellowish-pale. Tegulae, wing venation and pterostigma dark brown. Wing membrane uniformly slightly smoky, with iridescent sheen. Apical half of T1 to T3 brown amber, T4-T6 more darkened towards the apex, tending to brown. S1 to S4 yellowish-brown, the other sterna slightly darkened, tending to brown.

*Pubescence*: malar area covered with simple white setae, fairly dense, but extremely short. Face with relatively sparse setae, branched on base, decumbent at lower clypeus and parocular area, and semi-erect on forehead; setae of upper end of forehead with most compact branches. Erect setae intercalating the branched ones on face, whitish in the lower half and darkened a little above half; longest bristles on clypeus (0.1 mm) and vertex (0.2 mm), and shortest on middle and lower parocular areas (0.05 mm). Whitish setae on scape, with 0.025 mm. Metathoracic wings with five hamuli. Pro and mesothoracic trochanter with whitish simple hair on the underside; similar but more sparse on the metathoracic. Setae of legs darker from the apical third of femurs to the apex, also gradually longer and thicker towards of the legs apex. Disk of mesoscutum with erect and darkened setae, relatively long (0.25 mm) and thick, these even longer in posterior board of scutellum (0.3mm). Mesepisternum with simple, whitish and relatively long hairs (0.24 mm), interspersing the whitish and branched (0.12 mm). T1 and T2 practically glabrous with a narrow strip of lower and pale setae on their board, with some very short setae on the apical half of sides of terga, setae gradually thicker and darkened, from the widest apical band of T3 toward of the metasoma apex. Apical band especially wider in the middle region of terga; T6 with some simple setae interspersing branched setae; setae on T3 with 0.07 mm; T4 with 0.13 mm; T5 with 0.17 mm and T6 with 0.18 mm. S1–S6 with whitish setae on their apical bands, about 0.13 mm length, the last fully setaceous.

*Integument surface*: Fully smooth and shiny with only piliferous punctuation. Vertex distinctly higher at ocelli level.

*Measurements* (mm): Body length: 6.0. Head width: 2. Head approximately 1.5× wider than long (2.0: 1.34). Distance between the medium ocelli and the compound eye: 0.43; interorbital distances maximum and minimum (1.3: 0.91). Clypeus 1.7× wider than long (0.85: 0.48). Scape approximately 6× longer than its median width (0.61: 0.1). Pedicel about as long as wide (0.097: 0.096). Malar area: 0.05. Mesothoracic wing length of: 4.7. Metasoma elongated.

*Specimen condition*: very dirty, the left prothoracic tibia and tarsus, right prothoracic distitarsus, right mesothoracic tibia, tarsus and right metathoracic leg missing.

*Label data*: “Brasilia” [= Brasil], “631”, “365”, “Trigona latitarsis Friese, 1900” [written by Friese with black ink].

#### Description.

**Figures 13–18.**
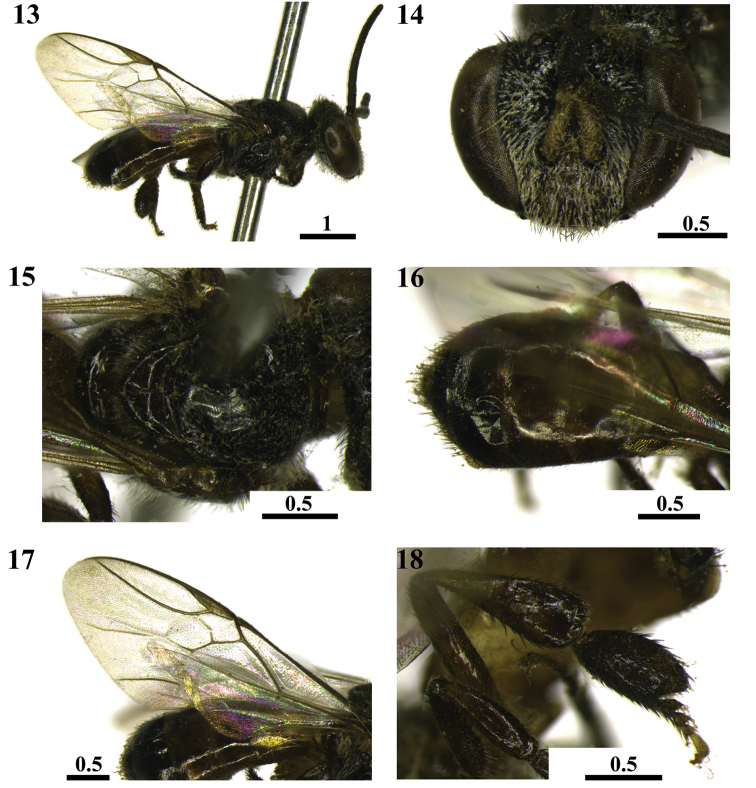
Lectotype male of *Trigona latitarsis* Friese, 1900 deposited at the Entomological Collection of the HNHM. Lateral view (**13**) Frontal view of head (**14**) Dorsal view of mesosoma (**15**) Dorsal view of metasoma (**16**) Forewing (**17**) Lateral view of hind leg (**18**). Scales in millimeters.

***Male* (lectotype) (Figs 13–18, 26).** *Color*: Integument predominantly brown-blackish. Clypeus brownish, labrum and apical third of mandible yellowish. Scape yellow with a brown spot in apical third of dorsal surface. Pedicel brown. Flagellum dark brown. Pronotal lobe translucid brown. Legs brown-yellow with brown darkened spots on the apical half of femurs, basitarsus more darkened than rest of legs; metathoracic tibia with large yellowish areas, with 1/5 apical and metathoracic board more darkened. The last tarsomeres yellow. Tegulae, alar venation and pterostigma brownish. Wings membrane uniformly slightly smoky, with iridescent sheen. T1 yellowish-brown, T2-T4 brown; T5 to T7 more darkened than the others. Sterna yellowish, the last two brownish.

*Integument suface*: similar to that of worker.

*Pubescence*: similar to the worker, except for the following characters: plumose pilosity densest on face; thinner and shorter setae on mesosome; setae of mesoscutum disc notably shorter, sparser and thinner, with few interspersed darkened setae; setae of the scutellum notably thinner and sparse, fully whitish; band of tiny setae on posterior board of T1 to T3 pale yellow; erect setae longer, thick and darkened from T4 to the tip of metasoma; erect setae on the sides of terga.

*Measurements* (mm): Body length: 5.00. Head width: 1.8. Head approximately 1.2× wider than long (1.8: 1.4). Distance between the compound eye and medium ocelli 0.5. Interorbital distance maximum and minimum (1.5: 0.7). Clypeus 1.6× wider than long (0.73: 0.45). Scape approximately 4× longer than its median width (0.56: 0.14). Pedicel 1.1× wider than long (0.17: 0.15). Malar area with 0.05. Mesothoracic wing length: 4.3. Metathoracic tibia 3.5× longer than wide (1.4: 0.4). Metathoracic basitarsus 2.1× longer than wide (0.84: 0.4). Metasoma elongated as the worker one.

*Lectotype conditions*: quite dirty, the left prothoracic leg and right metathoracic tibia missing.

*Label data*: written with black ink: “Brasil, S. Paulo”, “631/156”.

***Queen.*** Physogastric queen (Figs 21–24). *Color*: Integument predominantly brown yellowish. Clypeus yellowish brown with outlines margin darkened. Labrum and mandibles yellowish brown. Mandibular condyles blackened. Antennae yellowish. Scape yellowish brown. Pronotal lobes blackish brown. Legs brown yellowish. Upper surface of femurs slightly darkened. Apical third of the lower surface of femurs blackened. Basal and apical margin of tibiae blackish. Metathoracic tibia with blackish spot on external face next to the posterior border. Tarsus yellow with basal edges blackened. Tegulae, wing venation and pterostigma dark brown. Wing membrane hyaline. Apical half of T1 and T2 dark brown. T3 with the apical half brown. T4–T6 brown yellowish. S1–S4 brown with yellowish sides. S5 brown in lighter shade than the other sterna.

**Figures 19, 20.**
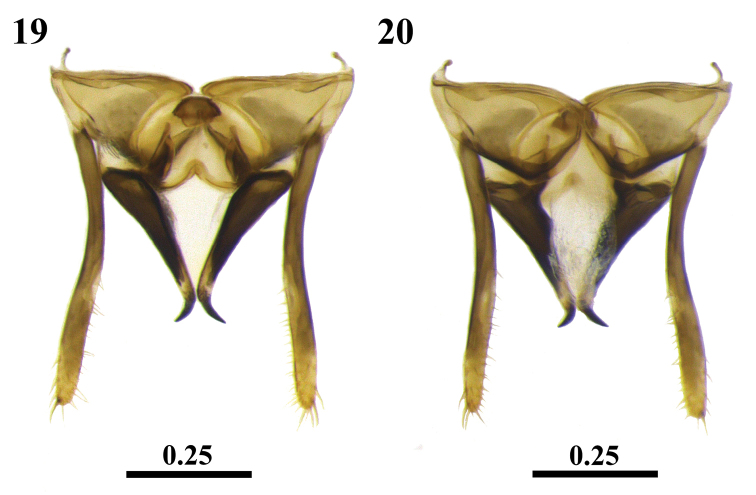
Male genital capsule of *Scaura latitarsis* (Friese, 1900). Dorsal view (**19**) Ventral view (**20**). Scales in millimeters.

**Figures 21–24.**
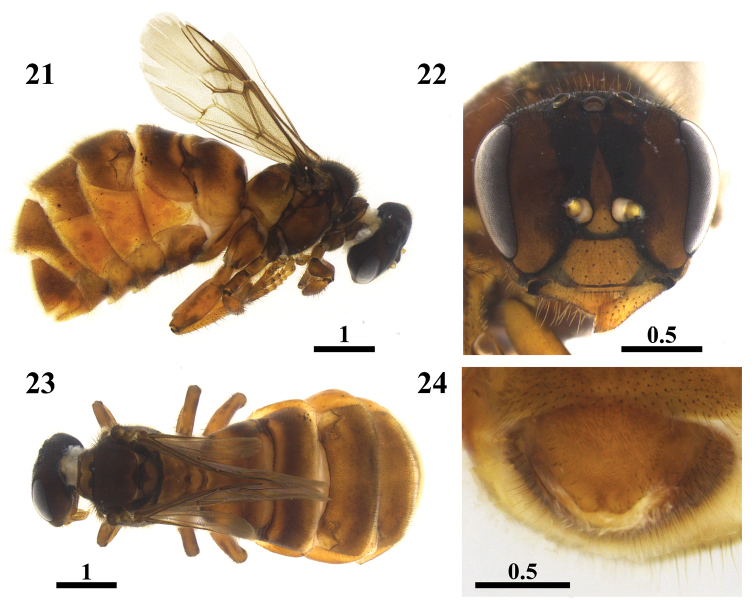
Physogastric queen of *Scaura latitarsis* (Friese, 1900). Lateral view (**21**) Frontal view of head (**22**) Dorsal view (**23**) Ventral view of fifth sterna (**24**). Scales in millimeters.

*Pubescence*: malar area with few whitish and short setae. Simple and short setae on upper half of head (0.05 mm). Long, thick and semi-decumbent setae on clypeus and labrum interspersing the simple, thin, and dark brown, the longer on apical half of clypeus (0.14 mm). Greater abundance of setae near the apex of mesothoracic wings, the metathoracic with 5 hamuli. Trochanters with simple whitish hairs which are more abundant on lower face. Basal third of prothoracic femurs and basal half of the mesothoracic with simple whitish hairs on the underside. Metathoracic femurs with longer setae on basal and latero-anterior range to the apical region. Metathoracic tibiae and basitarsus with yellowish setae with brownish sheen; on tibiae, setae longer in the posterior border. Disk mesoscutum with sparse and long blackish simple setae (0.19 mm), interspersing the very abundant whitish setae, these later with a third of the length of the largest blackish setae. Mesepisternum (0.20 mm) and metepisternum (0.12 mm) with only simple hairs. Anterior margin of T1 with simple sparse whitish hair. Apical margins of terga with blackish, short and less abundant setae throughout their length. Apical half of T2–T5 with dark setae. T6 with blackish setae throughout its length. Pubescence on T3 with 0.19 mm; on T4 with 0.23 mm; T5 with 0.25 mm and T6 with 0.23 mm. Apical half of S1–S4 with simple and whitish setae. S5 with setae in its entire length.

*Integument surface*: Head, meso and metasoma entirely smooth and shiny. Vertex slightly elevated. Tibiae and basitarsus smooth between microtrichia.

*Measurements* (mm): Body length: 6.15. Head width: 1.57. Head about 1.28× wider than long (1.57: 1.22). Distance between medium ocelli and the compound eye: 0.43; interorbital distances maximum and minimum (1.15: 1.07). Clypeus 2.38× wider than long (0.81: 0.34). Malar area 0.14. Length of metathoracic coxae, trochanters, femurs, tibiae and basitarsus 0.66: 0.41: 1.15: 1.47: 0.65, respectively. Metathoracic tibiae 5× longer than wide (1.47: 0.29). Metathoracic basitarsus 5.4× longer than wide (0.65: 0.12).

*Specimen condition*: good, with a loose antenna preserved in alcohol.

*Label data*: “*Scaura tenuis*, Nova Xavantina MT – 9/12, Rainha F., Mateus leg”, “Nova Xavantina, Nov/2012”, “Rainha *Scaura tenuis*”.

## Discussion

A male deposited in the HNHM is designated as the lectotype, which has a label with the following information: “Brasil, S. Paulo”, “631/156”.

**Figure 25.**
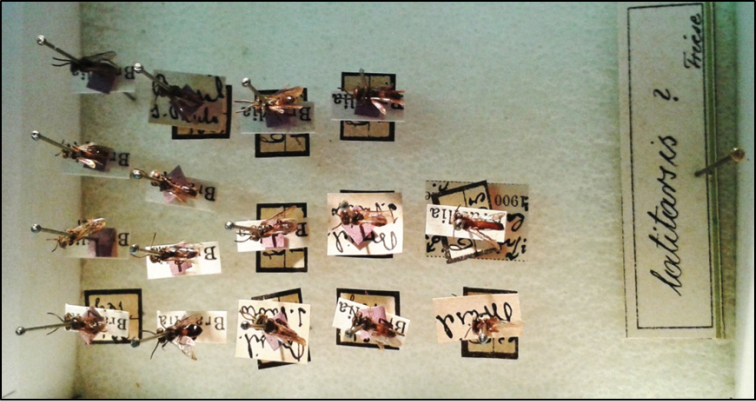
Syntypes of *Trigona latitarsis* Friese, 1900 deposited at the Entomological Collection of the HNHM.

A total of 15 paralectotypes (13 males and 2 worker) of *Trigona latitarsis* Friese, 1900 is also deposited in HNHM (drawer 78/47), which were studied, validated, and labeled by the second author (Fig. 25): five males, all with labels printed with the name “Brasilia” (corresponding to Brazil), and another label with the number “631/156” handwritten (one specimen also has a label written in black ink with Friese’s handwriting “*Trigona latitarsis* Friese, 1900”); six males with only a printed label with “Brasilia”; two males with a label handwritten in black ink, with the names "Brasil, São Paulo", also with another handwritten label with the number “631/156”; a worker with a handwritten label with the word “Brasil”, and another label handwritten and numbered “631/365”; a worker with a printed label in which is written “Brasil” and a handwritten label with the numbers “631/365”. Although the majority of syntypes of *Trigona latitarsis* deposited in the HNHM are males, and as Friese did not mention in the species description the females deposited at “Budapest collection”, there are no doubts that these two workers also belong to type series of the *Trigona latitarsis*, once the data confer with males labels (especially the number 631, which seems to be the number of the locality of collection – in this case "São Paulo" – or collector, as observed in *Trigona latitarsis* labels and also in the types labels of other species described by Friese in 1900 – same paper), all specimens are of the same species and had been kept separate together with males. It also important to mention that the putative type specimens are the only specimens of this species deposited at the HNHM. Probably, because they were among a large number of males, which was not usual for species of Meliponini described by him, Friese did not notice the two female specimens (therefore these two female were also marked as paralectotypes). Specimens from Suriname, mentioned by Friese (1900), were not found in the collections of Berlin, Vienna, Munich, Paris, or Budapest, which are collections where most of Friese’s types are deposited. All specimens in HNHM are labeled lectotype and paralectotypes by F. F. de Oliveira.

Material deposited in the ZMB: The worker, designated by Melo and Costa (2004) as lectotype of *Trigona latitarsis*, does not belong to the types series of this species according to our study, as this specimen has the following label data: “Brasil, Jundiahy, 1899, Schrottky”, “Zool. Mus. Berlin”, “latitarsis” [writing with blue ink pen], “LECTOTYPE, *Trigona latitarsis*, Friese, 1900, desig. Melo & Costa, 2004”. In his publication, Friese (1900) refers to Jundiaí (a town in São Paulo State, Brazil) in the description of other species, but not in the original description of *Trigona latitarsis*, where he cites only “São Paulo”. If the type locality was Jundiaí, he would have written the full reference of the label, as he did for the other species described in the same paper, also including the collector’s name (e.g. *Trigona schrottkyi* – page 386–387: “Brasilia: Jundiaby, 20. Oktoh. 1898 (Schrottky) und Saõ Paulo”). Unfortunately, all type series of *Melipona crassipes tenuis* Ducke, 1916 that should be in MPEG (Museu Paraense Emílio Goeldi, Pará, Brazil) are lost. Ducke (1916), in the description of this species, refers to specimens from Pará (Itaituba), Amazon (Rivers Javary and Japurá) and Mato Grosso (Tributaries of Tapajóz, collected by G. Kuhlmann). This fact had already been mentioned by Ducke (1916) when he described the geographical distribution of *Scaura latitarsis* (as “*Melipona latitarsis*”), where he clearly mentions that, according to Friese, this species occurs (or would be present) in São Paulo de Olivença (p. 28 lines 1 and 2). The true *Scaura latitarsis* has an elongated abdomen with abundant branched setae on the fifth and sixth terga. The species which has been interpreted as *S. latitarsis* by several authors for many years including Melo and Costa (2004) has short subtriangular abdomen (Oliveira et al. 2013), with few whitish hairs on the fifth and sixth terga.

It is noteworthy that all *Scaura* found in ZMB were examined and none of them correspond morphologically to *Scaura latitarsis*, nor do the label data correspond to those cited in the original description by Friese (1900). *Scaura latitarsis*: Melo & Costa, 2004 (unjustified lectotype).

*Scaura latitarsis* has been interpreted over the years by a number of different authors based on specimens from different regions of Brazil, including specimens from São Paulo State in southeastern Brazil, like the lectotype designated by Melo and Costa (2004). However, analyzing the older literature on this species and the type material of *Trigona latitarsis* (the newly designated lectotype and paralectotypes) deposited in HNHM and cited by Friese in the original description (16 specimens, two workers, and 14 male), we conclude that *Trigona latitarsis* does not correspond morphologically to what has traditionally been interpreted as *Scaura latitarsis*, and that these specimens that are traditionally (mis)interpreted as *S. latitarsis*. The species that have been traditionally (mis)interpreted as *S. latitarsis* (from São Paulo State and other regions of Brazil, as well as from Bolivia, Colombia, Guyana, Peru, Suriname and Venezuela), including the lectotype designated by Melo and Costa, corresponds to a new species (Nogueira *in litt.*).

Although the type material of *Melipona crassipes tenuis* Ducke, 1916 was not found, based on how species has been traditionally interpreted over the years by Ducke himself as *Trigona tenuis*, and also by Friese, Moure and Camargo, we conclude that the species described as *Melipona crassipes tenuis* Ducke, 1916 correspond to *Trigona latitarsis* Friese, 1900 (as junior synonyms) as interpreted in the current article. This conclusion is based on the type series of *T. latitarsis* and on the original description published by Ducke (1916), where he refers to the thin and elongated metasoma “Abdomen muito tenue” (p. 46), the main character that defines this species gaving rise its name, and which separates it from all the other species of *Scaura*.

The taxonomic confusion must have been generated by a misinterpretation of the type locality mentioned by Friese in the original description, cites only "Saõ Paulo", referring to São Paulo de Olivença city mentioned by Ducke (1916: 28), and not São Paulo State (southeastern Brazil). Given that *Scaura tenuis* does not occur in the state of São Paulo, but only in the Amazon region from Colombia, Ecuador, Peru, Suriname, French Guiana, Bolivia and Brazil (Fig. 29), we can conclude that the locality “Saõ Paulo” written on the label of some syntypes and mentioned by Friese in the original description refers to a small town in the Amazonas State, known as “São Paulo de Olivença”, and not the state of São Paulo, as it has been interpreted since 1900 by different Meliponini taxonomists, including Silveira et al. (2002), Camargo and Pedro (2002), Melo and Costa (2004), Camargo and Pedro (2013) and Oliveira et al. (2013).

**Figures 26–28.**
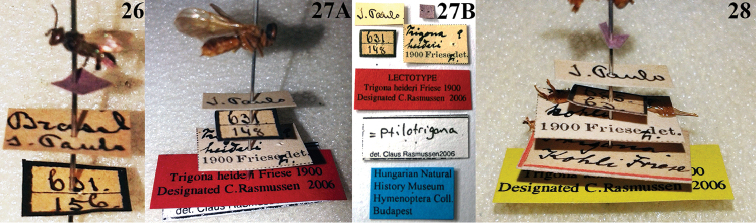
Labels of type material deposited at the Entomological Collection of the HNHM. Lectotype of *Trigona latitarsis* Friese, 1900 (**26**) Lectotype of *Trigona heideri* Friese, 1900 (**27A, B**) Paralectotype *Trigona kohli* Friese, 1900 (**28**).

**Figure 29.**
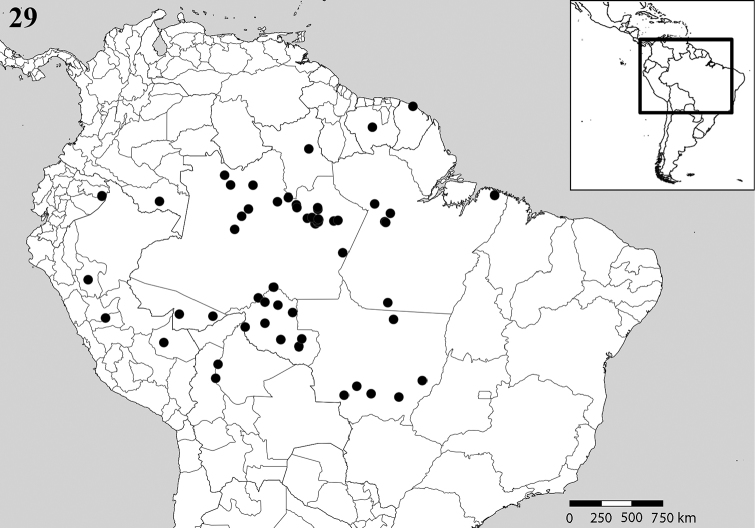
Map of South America showing the known geographic distribution records for *Scaura latitarsis* (Friese, 1900), based on the type material studied here, plus analysis of additional material analyzed from Amazon region and part of the Brazilian Cerrado; Bolivia: El Beni; Brazil: Acre, Amazonas, Pará, Rondônia, Roraima, Mato Grosso; Colombia: Amazonas, Meta; Ecuador: Napo; French Guyana: Cayenne; Peru: Huánuco, San Martín, Madre de Dios, Pasco; Suriname.

It is important to note that the Amazon locality now known as São Paulo de Olivença was founded as a village in 1689 (where a mission was carried out by Spanish missionaries), and, in this period, was known as “São Paulo Apóstolo”. However, in 1708, this village came under the dominion of Portuguese missionaries and had its name changed to “São Paulo dos Cambebas”. With its elevation to city status in 1817, it then received the name of “São Paulo de Olivença”. With the creation of the Solimões district, on June 13, 1884, São Paulo de Olivença became a well-known city in the region, for having been chosen as the administrative center of this new district. Thus, in 1900, when the species in question was described, the village was known and visited by researchers and religious people (BVA 2014). This definition of the type locality as São Paulo de Olivença is a very important fact, and may modify the interpretation of many of syntypes of different species described by Friese.

Another important and relevant fact, which provides strong support for this new interpretation of *Scaura latitarsis*, can be seen in the original description, when Friese describes the male exemplar as having yellow scape (Fig. 14). This is not the case in those male specimens of the species from São Paulo State that have been interpreted as *T. latitarsis* (1/3 basal is yellowish brown). Even if the material type of Friese (syntypes) comprised of specimens of two species (that were being interpreted individually and *S. tenuis* and *S. latitarsis*), the information provided by the original description leaves no doubt about the validity of the taxon correspondence that had been interpreted as “*S. tenuis*” as being the same as “*T. latitarsis*”, according to the original description (compared with syntypes studied here). Even if additional specimens belonging to the syntypes series be found in the future, and these prove that the “*T. latitarsis*” type series is composed of more than one species, it would not invalidate the new lectotype from Budapest (HNHM), as Friese clearly included males from Budapest Museum in the type series.

Friese emphasized in the original description that the yellow scape occurred only in the male. However, the ventral side of the scape is also yellow, in workers, although the color is restricted to the basal third. As the male scape is completely yellow with a strong tonality, it may have been this difference which Friese wished to emphasize in his description. Consequently, all specimens must have been collected in São Paulo de Olivença, although it was written on the label only “S. Paulo” (as noted in specimens deposited in HNHM). Additional important evidence comes from the fact that Friese does not mention Jundiaí in the description, as he does for other species described in the same article, and whose type material type was collected in Jundiaí (e.g. *Trigona schrottkyi* – pages 386–387 as mentioned previously). Therefore, the only encountered specimens found that we are confident belong to the type series are those of the HNHM, based on the description and data of the labels affixed to the specimens.

A similar case occurs with the Amazonian species *Trigona heideri* Friese, 1900 (= *Ptilotrigona lurida* (Smith, 1854)) described by Friese under the number 39, in the same paper (pages 389 - 390), one page after he described *Trigona latitarsis* Friese, 1900 (number 34, page 388). The species *Trigona heideri* was described by Friese based on 14 workers from Brazil (Brasilia: Para (Ducke, 29. Septbr. - 11. Novbr. 1899); S. Paulo, Obidos, Fonteboa, Amazonas), Columbia and Peru (Vilcanota), and the specimens were also deposited also at the HNHM.

Based on the type material from HNHM the specimen chosen by Claus Rasmussen (27.III.2006, *in litt.*), and designated as lectotype of *Trigona heideri* Friese, 1900, is a specimen from “S. Paulo”, and, like *Trigona latitarsis* Friese, 1900 has the same handwritten label in black ink (with the same handwriting) (Fig. 27A, B). For this species, Camargo and Pedro (2013) had also assumed that the type locality "S. Paulo" is in fact São Paulo de Olivença (Amazonas, Brazil), as this is an Amazonian species, and one that has never been recorded in São Paulo State (Southeastern Brazil).

An additional and similar case with *Trigona kohli* Friese, 1900 (=*Trigona pallens* (Fabricius, 1798)), described by Friese (1900) in the same article (number 29 on page 387), whose paralectotype was designated by Claus Rasmussen from the HNHM type series (Fig. 28). This too is an Amazonian species never recorded in São Paulo State (southeastern Brazil).

Carefully comparing the provenance labels of *Trigona latitarsis* Friese, 1900 (here designated as lectotype) (Fig. 26), *Trigona heideri* Friese, 1900 (lectotype designated by Claus Rasmussen) (Fig. 27 A, B) and *Trigona kohli* Friese, 1900 (paralectotype designated by Claus Rasmussen) (Fig. 28), it is easily seen that the three specimens have the same handwritten label in black ink, written by the same letter, which leads us to assume that they were collected in the same locality and had the labels written by hand by the same person. Also the number 631 appears on observed in the label of *Trigona latitarsis* (Fig. 26) and *Trigona heideri* (Fig. 27 A, B), which seems to be the some information about the locality of collection (in this case “São Paulo”), date, or collector.

Based on all information discussed here, we propose the male specimen in HMHN carrying the label written “S. Paulo” to be considered as lectotype of *Trigona latitarsis* Friese, 1900, replacing the specimen from Jundiaí designated by Melo and Costa (2004) (lectotype invalidated) and *Melipona crassipes tenuis* Ducke, 1916 and are to be considered a junior synonym of *Trigona latitarsis* Friese, 1900.

### Summary of arguments for the current interpretation

1. Ducke (1916, p. 28, lines 1 and 2) received information from Friese himself and he said that *Trigona latitarsis* Friese, 1900 was from São Paulo de Olivença (a village in Amazon) and not from São Paulo State (Southeastern Brazil);

2. The label of *Trigona latitarsis* Friese, 1900 type material had written only the word São Paulo (village), as discussed in here. This is also the case for several other types from a number of different species described by Friese (1900), for which we had the opportunity of study the types in the HNHM;

3. The word “S. Paulo” without any other information ended up causing confusion as it referred to a village in Amazon and not to São Paulo State, which is what came to be assumed, even though this locality is situated in another, completely different, region;

4. There is no evidence from identification labels, or any other information associated with original description that proves that the specimen chosen by Melo and Costa (2004) could be, at least, be considered syntypes of *Trigona latitarsis* Friese, 1900;

5. Currently, the only material that can, for certain, be considered as syntypes of *Trigona latitarsis* Friese, 1900 is the material deposited at the HNHM and discussed here;

6. Even if the type series was composed of multiple species, as observed for several species described by Friese, and other material from Brazil or Surinam could be found in the future, and if it represented a different taxonomic entity (species), this does not invalidate the types from the Budapest Museum (HNHM), as once Friese clearly included these as syntypes of *Trigona latitarsis* Friese, 1900;

7. The same situation as occurred with *Trigona latitarsis* Friese, 1900, has also occurred with *Trigona heideri* Friese, 1900, where the same label with the same Indian ink handwriting was found. It is from this that Camargo and Pedro Pedro (2013) once again assumed that the locality to be São Paulo de Olivença and not São Paulo State (Southeastern Brazil);

8. This situation was also observed in *Trigona latitarsis* Friese, 1900, with the same label with the same label with identical handwriting in Indian ink was foud for *Trigona kohli* Friese, 1900 (=*Trigona pallens* (Fabricius, 1798)), which again is an purely Amazonian species and does not occur in São Paulo State (Southeastern Brazil);

9. In the same article win which Friese (1900) described *Trigona latitarsis* and several other species, the species from Jundiaí (Ex: *Trigona schrottkyi* Friese, 1900) has the following information on the label: Brasilia: Jundiaby [sic = Jundiaí, São Paulo], 20.X .1898, Schrottky leg., as observed in type. Why would Friese suppress this locality information only for *Trigona latitarsis*?

10. Some specimens of the syntype series from HNHM discussed in here have a Friese identification label, handwritten by that author (partially or totally with Indian ink), except that designated by Melo and Costa (2004).

11. Although the type material of *Scaura tenuis* was not found (is having been lost), in the original description by Ducke (1916), he refers to the slender metasome “Abdomen muito tenue” (p. 46), which supports the synonimization of *S. tenuis* under *S. latitarsis*.

## Supplementary Material

XML Treatment for Scaura latitarsis
